# Applying in-situ visible photopolymerization for fabrication of electrospun nanofibrous carrier for meloxicam delivery

**DOI:** 10.1038/s41598-023-36893-9

**Published:** 2023-06-16

**Authors:** Z. Ahmadipour, M. S. Seyed Dorraji, H. R. Ashjari, F. Dodangeh, M. H. Rasoulifard

**Affiliations:** Applied Chemistry Research Laboratory, Department of Chemistry, Faculty of Science, University of Zanjan, Zanjan, Iran

**Keywords:** Materials chemistry, Polymer chemistry, Nanoscale materials, Nanoscale materials

## Abstract

Despite meloxicam’s many benefits, it will cause many drawbacks if the meloxicam release rate is not controlled. Accordingly, we introduced a technique based on the electrospinning process to control the release rate and also to reduce side effects. For this purpose, different nanofibers were used as drug couriers. Nanofibers were prepared using polyurethane, polyethylene glycol, and light curable poly (ethylene glycol) diacrylate (PEGDA) by electrospinning. In fact, light curable poly (ethylene glycol) diacrylate (PEGDA) was synthesized as a hydrophilic functional group. Next, PEGDA and polyurethane were used simultaneously to fabricate the drug carrier nanofiber in a single processing step, and the electrospinning apparatus was equipped with a blue light source for in-situ photopolymerization during the electrospinning process. The molecular structures of nanofibers and PEGDA were investigated by FT-IR, ^1^H NMR, ^13^C NMR, SEM, TEM, XRD, and DSC analyses. Finally, we reduced in vitro drug release to 44% within ten hours, while the minimum release of meloxicam from the tablet was 98%.

## Introduction

Meloxicam is a non-selective, non-steroidal and anti-inflammatory drug (NSAID). In fact, is used as a treatment for rheumatoid arthritis, osteoarthritis, and ankylosing spondylitis^1,2^. According to studies, meloxicam oral administration has shown analgesic and anti-inflammatory activities in many scientific sources. However, many adverse side effects have been reported for this class of drugs^2,3^. Indeed, hypophyse and hypothalamus suppression, cardiovascular and renal failure, gastrointestinal bleeding, water and salt retention, hypophyse and hypothalamus suppression, and osteoporosis are some meloxicam side effects. Therefore, we need a high-performance drug delivery system to control the administered dose and its side effects^2,3^.

As studies have shown, electrospinning is a versatile and simple method to fabricate micro and nanofibers in different forms. Additionally, synthetic and natural polymers and hybrid materials can be electrospun into micro and nanofibers^4,5^. Electrospinning techniques possess numerous usages in the manufacturing of filters, soft tissue prosthetics, tissue engineering scaffolds, reinforced composites, porous electrodes for battery separators, protective garments, and controlled drug delivery^6^. Indeed, the science of drug delivery systems (DDS) has dramatically developed in recent years, especially by using electrospinning techniques^7,8^. It is worth mentioning that one of the advantages of these techniques is the production of highly porous fibers, which increases their surface-to-volume ratio and can improve drug loading^7,8^. In addition to the high surface-to-volume ratio of fibers, unlike other techniques, such as encapsulation by using the electrospinning technique in DDS designing, therapeutic compounds can be conveniently embedded within polymeric carriers^9^. Also, the electrospinning technique could be used for the preparation of nanofibers in a green or environmentally friendly way as well as biocompatible one using natural solvents that could be a potentially beneficial technique for the development of novel and bio-friendly drug delivery systems based on nanofibers^10^.

There are three electrospinning methods to prepare electrospun fibers, including blend, emulsion, and coaxial methods which can also be used for DDS designing. Due to the fact that most drugs prefer to disperse near the fiber surface or on the surface, therefore the fibers have an intense explosive release in the initial stage during the blend electrospinning process. Accordingly, to overcome this problem, a core/shell structure for fibers is recommended. Therefore, these fibers are prepared using emulsion and coaxial electrospinning methods. In fact, in core/shell systems, drugs are loaded into the core structure and the outer polymer (shell) acts as a barrier^11,12^.

Polymers that contain molecules physically or chemically entrapped in them play an essential role in modern pharmaceutical technology^13,14^. Among various polymers, polyurethanes (PUs) are versatile polymers; they often have biomedical applications, especially in DDS^15,16^. PU_S_ possess good elasticity and unique mechanical properties, additionally they are more biocompatible compared to other synthetic polymers. However, due to poor hydrophilicity and hemocompatibility, the use of PU_S_ in tissue engineering is restricted. As studies show, the scaffolds should have an excellent hydrophilicity to show good performance, and therefore PUs should undergo surface modifications to fulfill the desired needs^17,18^. Polyethylene glycols (PEGs) possess unique properties such as high hydrophilicity, low toxicity, and good biocompatibility, which makes them a proper candidate for biomedical applications, and they are mainly utilized for the modification of biomaterials^19,20^.

On the other hand, high hydrophilicity lead to PEG scaffold dissolving and explosive drug release in buffer solution. To overcome the problem, the PEG carrier structure should be crosslinked and turned into a network structure^21^. Dehydration is one of the most critical methods to crosslink these polymers^22^. However, this method is time-consuming and during the process, not only PEG faces dissolving and destruction, but the drug might also undergo crosslinking reaction. Another method that can be used for crosslinking is light-induced polymerization. The light-induced polymerization is a promising technique due to its low cost, fast reaction rate, simple equipment, and sustainability^23^.

In the current study, attempts were carried out to prepare drug carriers to control the release rate of meloxicam and prevent it from explosive release. Accordingly, different nanofibers, such as monolithic, blend, and core/shell nanofibers were synthesized through the electrospinning technique by using PU and PEG polymers. On the other hand, light curable poly (ethylene glycol) diacrylate (PEGDA) was used to prevent the PEG scaffold from degradation, and for the fabrication, in-situ photocrosslinking was used during the electrospinning process. Besides, the chemical structure, morphology, and properties of nanofibers and PEGDA were characterized using ^1^H NMR, ^13^C NMR, FT-IR, SEM, TEM, XRD, and DSC analysis methods. Finally, the performance of nanofiber drug carriers was studied in phosphate-buffered saline (PBS) solution.

## Experimental section

### Materials

Polyurethane (PU, Mw = 110,000), Polyethylene glycol (PEG, Mw = 10,000), magnesium sulfate (MgSO_4),_ dimethylformamide (DMF), tetrahydrofuran (THF), chloroform, dichloromethane, triethylamine, sodium chloride and sodium hydroxide were purchased from Merck Co. Camphorquinone, acryloyl chloride (97.0% purity), N,N-dimethylaminoethyl methacrylate, disodium monohydrogen phosphate, potassium dihydrogen phosphate, ammonium chloride, meloxicam, Tween 80, and potassium chloride were obtained from Sigma Aldrich Co. Phosphate-buffered saline (PBS) tablets was purchased from Zist Mavad Pharmed.

### Preparation of electrospun solutions

#### Monolithic PU fiber

In order to prepare PU fibers, PU (8% w/v) solution was prepared in THF/DMF (1:1, v/v), and 0.1 percent of the drug was added to it. After that, PU fibers were then prepared through electrospinning with electrospinning parameters mentioned in Table 1.

**Table 1:** Electrospinning parameters.

	Monolithic fiber	Core/shell	Core/shell	Blend	Core/shell
Polymer	PU	PU/PU	PEG/PU	PEG–PU	PU/PEGDA
Voltage (KV)	22	22	15	14	15
Distance (mm)	200	200	200	200	200
Rate (ml/h)	1.8	0.9	0.3	0.6	0.3
T (^o^C)	30	30	30	30	30

#### Coaxial electrospinning of PU/PU nanofibers

Preparing core/shell PU/PU nanofibers included the following steps, first two identical solutions of PU (8% w/v) in THF/DMF (1/1, v/v) were prepared. One of these solutions was selected as the core solution and 0.1% of the drug was added to it, while the shell solution did not contain the drug. Following this, the nanofibers were electrospun, using coaxial electrospinning with factors listed in Table 1. To collect the fiber, a rectangular aluminum foil (20 * 20 cm) was utilized.

#### Coaxial electrospinning of PEG/PU nanofibers

PEG/PU nanofibers were prepared through the following steps. Firstly, the core solution of PEG (15% w/v) in chloroform/DMF (7:3, v/v) was prepared and 0.1% of the drug was added. Next, for preparing the shell solution, PU powder was dissolved in THF/DMF (1:1, v/v) and PU (8% w/v) was gained. Subsequently, the final solution was electrospun using the method mentioned in Table 1.

#### Preparation of blend PU/PEGDA nanofibers

For this step, a solution containing PU (8% w/v) and PEGDA (15% w/v) in THF/DMF (1/1, v/v) was prepared and 0.1% of the drug was added. The fiber was then electrospun through factors provided in the Table 1 at the temperature of 30 °C.

#### Synthesis and characterization of poly (ethylene glycol) diacrylate with average Mn = 10,000 (PEGDA)

**Figure 1:**
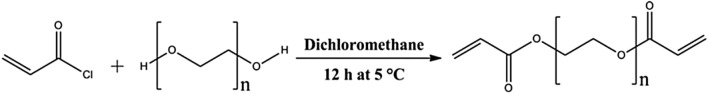
Synthesis of PEGDA.

PEG (5.00 g, 0.5 mmol) was dissolved in 60 mL of distilled dichloromethane. Triethylamine (2.1 ml, 5 mmol) was added to the reaction mixture and followed by stirring under a nitrogen purge for 30 min. Acryloyl chloride (0.11 g, 1.2 mmol) was dissolved in 5 mL of distilled dichloromethane and added slowly via an addition funnel to the reaction mixture. This reaction mixture was stirred for 12 h at 5 °C and stored under a nitrogen blanket, in darkness to avoid any hydrolysis and ambient photoreactions. Next, for further purification, the resulting solution was washed with a saturated solution of ammonium chloride, and drying by MgSO_4_, a colorless viscous liquid (95%) was obtained. The reaction is depicted in Fig. 1.

^1^H NMR, ^13^C NMR, and FT-IR spectroscopy measurements were used to PEGDA analysis. ^1^H NMR and ^13^C NMR spectra were recorded using a Varian Unity 250 MHz spectrometer in D_2_O. Also, chemical structure of PEGDA was studied by using a Fourier transform infrared spectrophotometer (FTIR, Perkin-Elmer) in a region of 400–4000 cm^−1^ and with the resolution of 4 cm^−1^.

#### Coaxial electrospinning of PU/PEGDA nanofibers

For this purpose, camphorquinone as visible light photo-initiator (3% w/w) and N,N-dimethylaminoethyl methacrylate as Co-initiator (0.5%, w/w) were first added to the obtained PEGDA (mixture A). Then the solution of mixture A (0.2% w/v) in Chloroform/DMF (7:3, v/v) was prepared as the shell solution. All the above steps were performed in darkness to avoid any ambient photoreactions.

For the core solution, a solution of PU (8% w/v) was prepared in THF/DMF (1:1, v/v), and 0.1% of the drug was added to it.

To prepare a homogeneous solution, all the above solutions were stirred over 12 h at an ambient temperature. Finally, the fibers were electrospun through factors provided in the Table 1 at the temperature of 30 °C and under visible light.

### Electrospinning

Each solution prepared in the previous steps was poured into a plastic syringe with a steel capillary of 0.69 mm diameter for the electrospinning. The solutions were supplied by a syringe pump, and the flow rate was maintained using a syringe pump. The distance of the nozzle to the collector was set at 20 cm and an aluminum foil with the dimensions of 20 * 20 cm^2^ was used as a collector. The electrospinning was performed to prepare monolith polymeric fibers, blend fibers, and core/shell fibers. Monolithic (pure PU) and blend PU/PEGDA solutions were electrospun by one needle supplying only one solution, but the core/shell solutions were fed to the spinnerets using two syringe pumps. A defined voltage is applied between the electrospun nozzle and collector using a high-voltage power supply (BGG4-21, BMEI Co., Ltd). The visible light was irradiated directly on the solution while it was coming out of a syringe and collected on the foil, and the syringe was covered to prevent the inner solution from polymerization. Other conditions of electrospinning are shown in Table 1.

### Swelling degree

The swelling degree (SD) of the nanofibers was determined by immersing the samples (0.05 g) in the PBS (pH:7.4) at 37 °C for different time intervals (4, 12 and 24 h), which was then followed by withdrawing the nanofibers, and weighting them. Next, Swelling Degree was calculated according to equation (Eq. 1):

1 $$\mathrm{SD }(\mathrm{\%}) = \left(\frac{\mathrm{Ws}-\mathrm{Wd }}{\mathrm{Wd}}\right) \times 100$$

where Ws is the weight of swollen nanofiber and W_d_ is the weight of dry nanofiber. The experiments were performed in triplicate.

### Characterization

The surface morphologies of the nanofibers were studied by a scanning electron microscope (SEM, NoVaTM Nano SEM 430, FEI Co.) using an accelerating voltage of 1 kV and a secondary-electron detector**.** Prior to the observation, all nanofibers were vacuum covered with a thin layer of gold. The average diameter of fibers was investigated by image J analysis software (Image-Pro Plus 6.0, USA) in the SEM photos. For this purpose, 100 fibers were randomly selected to measure the average diameter using image J software. The chemical structures and interactions of obtained polymers and nanofibers were studied by a Fourier Transform Infrared Spectrophotometer (FTIR, Perkin-Elmer) in the region of 400–4000 cm^−1^ with a resolution of 4 cm^−1^. Transmission electron microscopy (TEM) technique was used to confirm the core/shell structure of the PU/PEGDA fiber. To investigate the physical state of pure meloxicam and nanofibers, the DSC analyses were performed using a Mettler-Ms603s differential scanning calorimeter. In fact, an approximately 5 mg sample was weighed into an aluminum pan and covered with a lid having a 50 mm pinhole and all the samples were analyzed from 10 to 310 °C with a heating rate of 10 °C/min. Additionally, measurements were performed in an inert nitrogen flow (50 mL/min).

The crystalline structure of samples was characterized using an X-ray diffraction pattern. The X-ray diffraction pattern of the samples was obtained using a diffractometer (XRD-7000, Shimadzu). ^1^H NMR and ^13^C NMR spectroscopy studies were used for PEGDA analysis. ^1^H NMR and ^13^C NMR spectra were recorded using a Varian Unity 250 MHz spectrometer in D_2_O.

### In vitro drug release

In order to study drug release in nanofibers, the following steps were taken. First, the fibers were separated from the aluminum foil with caution. They were then kept in a vacuum for 12 h. Next, the samples containing 0.005 g drug were placed into 200 ml of PBS (pH 7.4) at the temperature of 37 °C with a stirring rate of 100 rpm. Subsequently, at certain time intervals, 4 ml of sample solution was withdrawn and replaced with 4 ml of fresh BPS solution. Following this step, the withdrawal solution was detected by UV–Vis spectrometry at λ = 361 nm [pardini2015 35]. Each experiment was repeated three times, and the amount of drug released to the PBS solution was calculated using (Eq. 2):

2 $$Cumulative \; percentage \; release (\%)={P}_{t}+{\left({P}_{t-1}\right)}^{V_{1}/V_{t}}$$

In the above-mentioned equation, P_t_ and P_t–1_ are the release percentage at time t and release percentage before “t” respectively. Additionally, V_t_ and V_1_ are the total volume of the system and withdrawn volume, respectively.

### Kinetics studies

Concerning optimum conditions for a drug delivery system, it is necessary to determine the mechanism controlling drug release. There are four models to identify the controlling mechanism including, the zero order, first order, Higuchi, and Korsmeyer-Peppas, which are used in this study. They could be shown as below:

Zero or model:

3 $${Q}_{t}={k}_{0}t$$

First order model:

4 $$LogQ=Log{Q}_{0}-\frac{{k}_{1}t}{2.303}$$

Higuchi model:

5 $$Q= {k}_{H}{t}^{1/2}$$

In these equations, *k*_*0,*_* k*_*1*_, and *k*_*H*_ are considered as the drug release rate constants of zero order, first order, and Higuchi models, respectively; Q is the amount of drug, released over a specific time (*t*).

Korsmeyer-Peppas model:

6 $$\frac{{M}_{t}}{{M}_{\infty }}=k{t}^{n}$$

In the abovementioned equation, *k* is the release rate constant, *M*_*t*_***/M***_*∞*_ is defined as the fraction of drug released from the hydrogel in a specific time (*t*), and finally, “n” is the diffusion exponent, based on “n” value, the controlling mechanism is determined (please see Table 2). In fact, Korsmeyer-Peppas is mainly utilized for polymeric systems.

**Table 2:** Explanation of diffusional release mechanism based on the value of ‘‘n’’.

Release exponent (n)	Drug transport mechanism
n = 0.45	Fickian diffusion
0.45 < n < 0.89	Non-Fickian transport
n = 0.89	Case II transport
n > 0.89	Super case II transport

## Result and discussion

### Investigation of PU fibers’ morphologies

**Figure 2:**
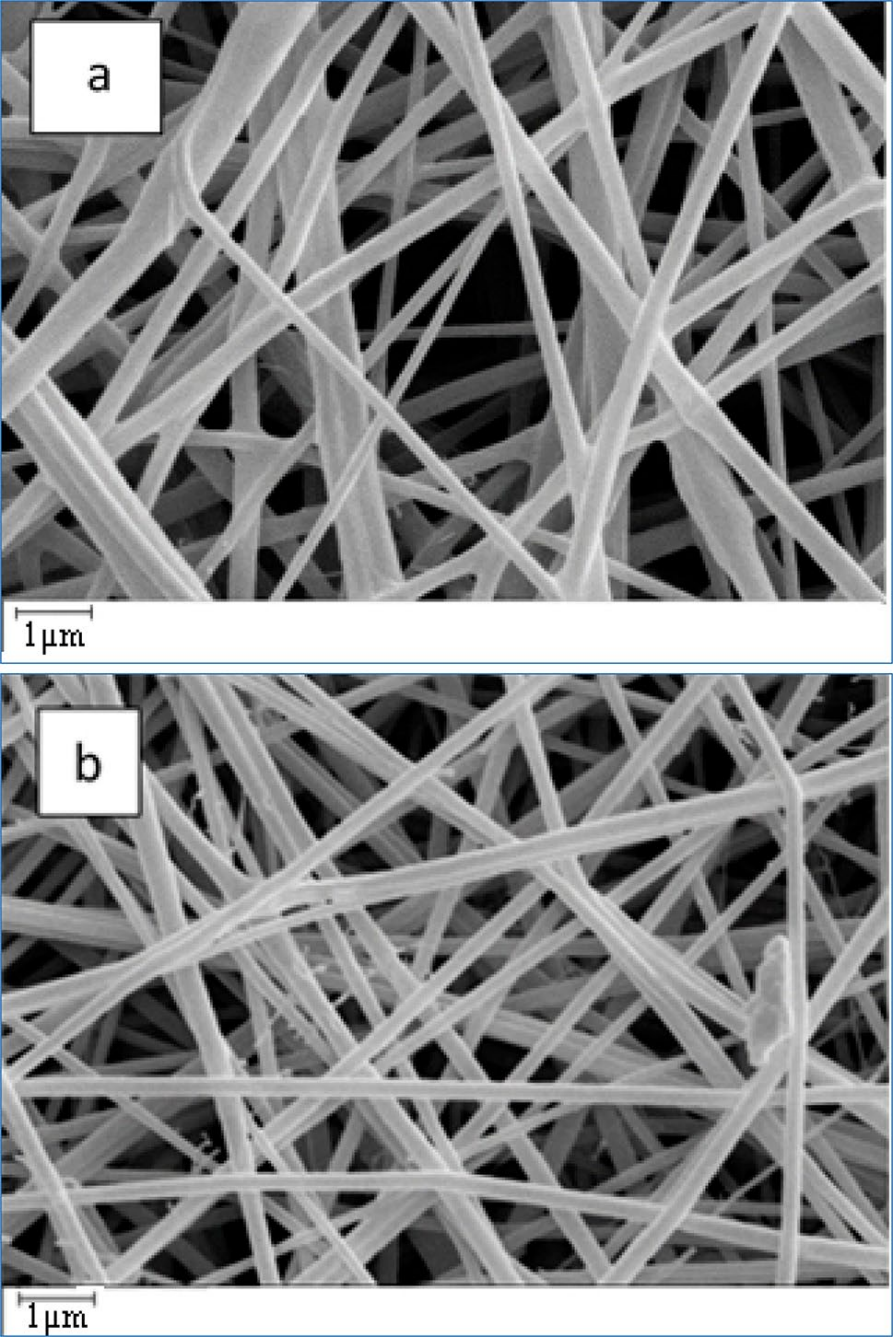
SEM images of (**a**) monolithic PU fiber and (**b**) core/shell PU/PU fiber.

The morphologies of prepared monolithic PU fiber and core/shell PU/PU were specified using SEM analysis (see Fig. 2a,b). As it could axiomatically be observed, the obtained fibers have a smooth surface without the noticeable beads.

### Study of PU fiber and drug interaction

**Figure 3:**
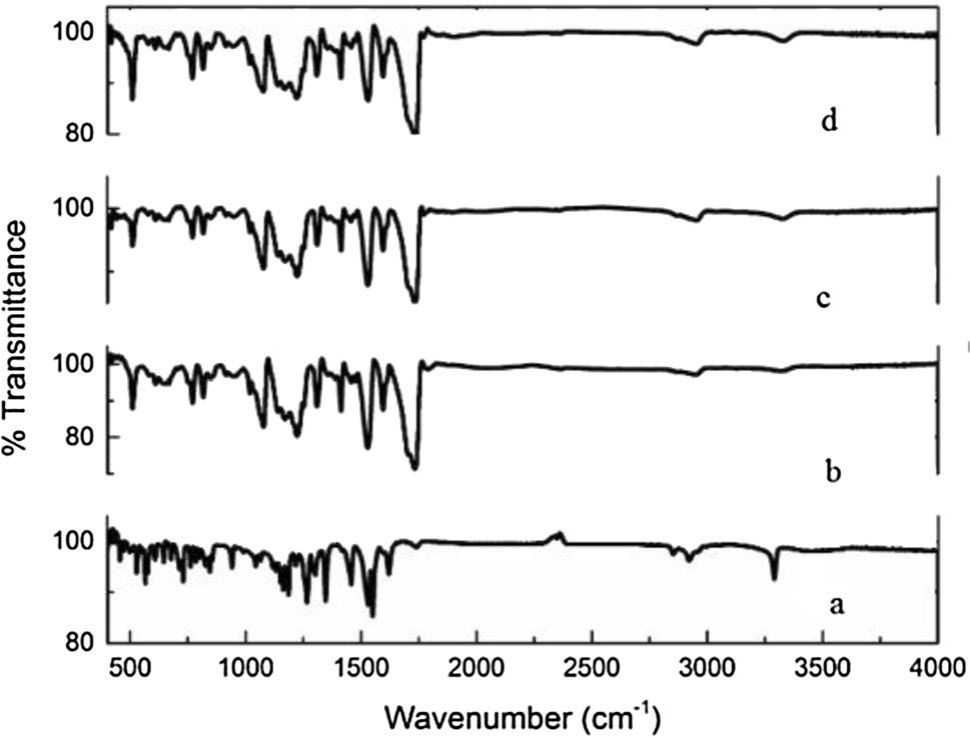
Analysis of chemical structure of (a) meloxicam, (b) pure PU nanofiber, (c) PU nanofiber with drug, (d) core/shell PU/PU nanofiber with drug.

In order to confirm drug loading into the PU fiber structure, and their interaction, FTIR spectra for meloxicam, pure monolithic PU fiber, monolithic PU fiber with the drug, and core/shell PU/PU nanofiber with drug were compared. FTIR spectrum of meloxicam (Fig. 3a) shows two peaks at 3290 cm^−1^, and 1153 cm^−1^ due to the stretching vibrations of N–H and S=O of meloxicam, respectively. Besides, (Fig. 3b) shows the FTIR spectrum of pure monolithic PU fiber, in which peaks at 3321 cm^−1^, 2954 cm^−1^, 1222 cm^−1^, and 1732 cm^−1^ are associated with stretching vibrations of N–H, C–H, C–C and C=O, respectively. Furthermore, looking at the FTIR spectrum of monolithic PU fiber with drug (Fig. 3c) shows a similarity to the spectrum of drug-free nanofibers, which means peaks of nanofibers and the drug overlapped. Therefore, it indicates a lack of chemical reaction between polymer and drug. However, it is worth mentioning that due to low amount of the drug (0.1%) in fibers, it might not be detected by FT-IR analysis. Moreover, the FTIR spectrum of core/shell PU/PU nanofiber (see Fig. 3d) and monolithic PU fiber are similar.

### Study of meloxicam release from monolithic PU and core/shell PU/PU nanofibers

**Figure 4:**
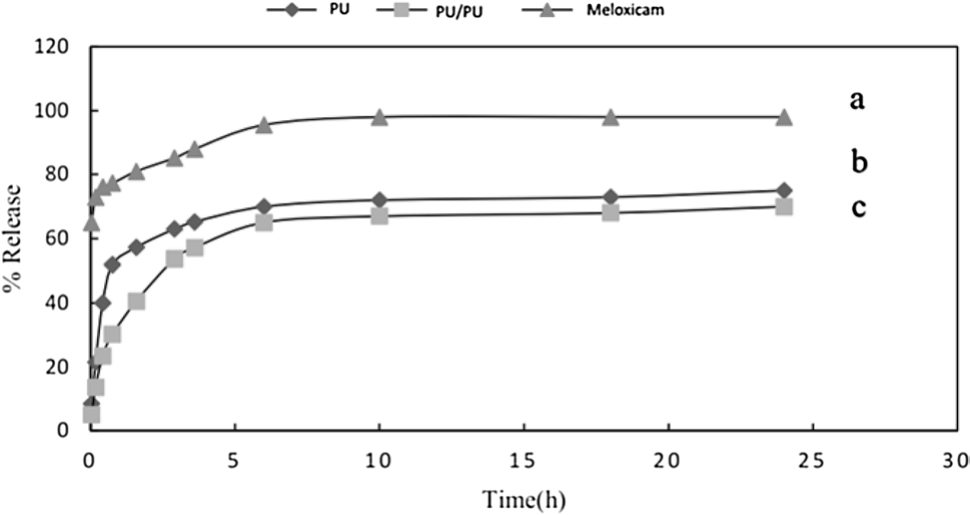
In vitro drug release profile of (a) meloxicam, (b) meloxicam from monolithic PU and (c) meloxicam from core/shell PU/PU nanofibers.

The rate and amount of drug release from the nanofibers structure were evaluated using PBS buffer (pH 7.4). For this purpose, the PU nanofibers containing the drug were put into a tea bag and transferred into 200 mL of PBS buffer at the temperature of 37 °C and stirred with the speed of 100 rpm. As it could axiomatically be observed in (Fig. 4), the core/shell PU/PU nanofibers show a controlled release compared to monolithic PU nanofibers. Regarding the core/shell structure of PU/PU nanofibers, it can be effectual on the release of meloxicam by decreasing the rate of buffer diffusion to the nanofiber and drug release from the nanofiber structure, while monolithic PU nanofibers experienced a faster release.

### Study of release kinetics for PU nanofibers

In order to investigate and identify the mechanism of meloxicam release and kinetics of the controlling mechanism, different models, such as the zero order, first order, Higuchi, and Korsmeyer-Peppas models were utilized. For this purpose, drug release was investigated for monolithic PU and core/shell PU/PU nanofibers over 45 min and from 45 min to 6 h. Indeed, the data from the study were fitted and evaluated based on the correlation coefficient R^2^. Table S1, Figs. S1 and S2 show the obtained kinetic parameters and models for monolithic PU and core/shell PU, respectively (please see Table S1, Figs. S1 and S2 in supplementary data).

As results show for monolithic PU, there is a close fit to the Korsmeyer-Peppas model with correlation coefficient (R^2^) of 0.9976 and 0.9986 for first 45 min and the time from 45 min to 6 h, respectively, whilst other models indicate poor fit due to lower R^2^. Besides, according to the value of “n” it can be concluded that the release of meloxicam experienced the non-Fickian mechanism for first the 45 min and the Fickian mechanism after 45 min. Concerning core/shell PU, it shows a close fit to the First order model with a correlation coefficient (R2) of 0.9956 for the first 45 min, while after 45 min to 6 h, it experienced a close fit to the Korsmeyer-Peppas model with a correlation coefficient (R^2^) of 0.9823. Moreover, as regards the mechanism controlling drug release, meloxicam experienced a non-Fickian mechanism for the first 45 min and a Fickian mechanism after 45 min to 6 h with the “n” value of 0.5772 and 0.382, respectively (please see Table S1, Figs. S3 and S4 in supplementary data).

### Investigation of PEG/PU nanofibers’ morphology

**Figure 5:**
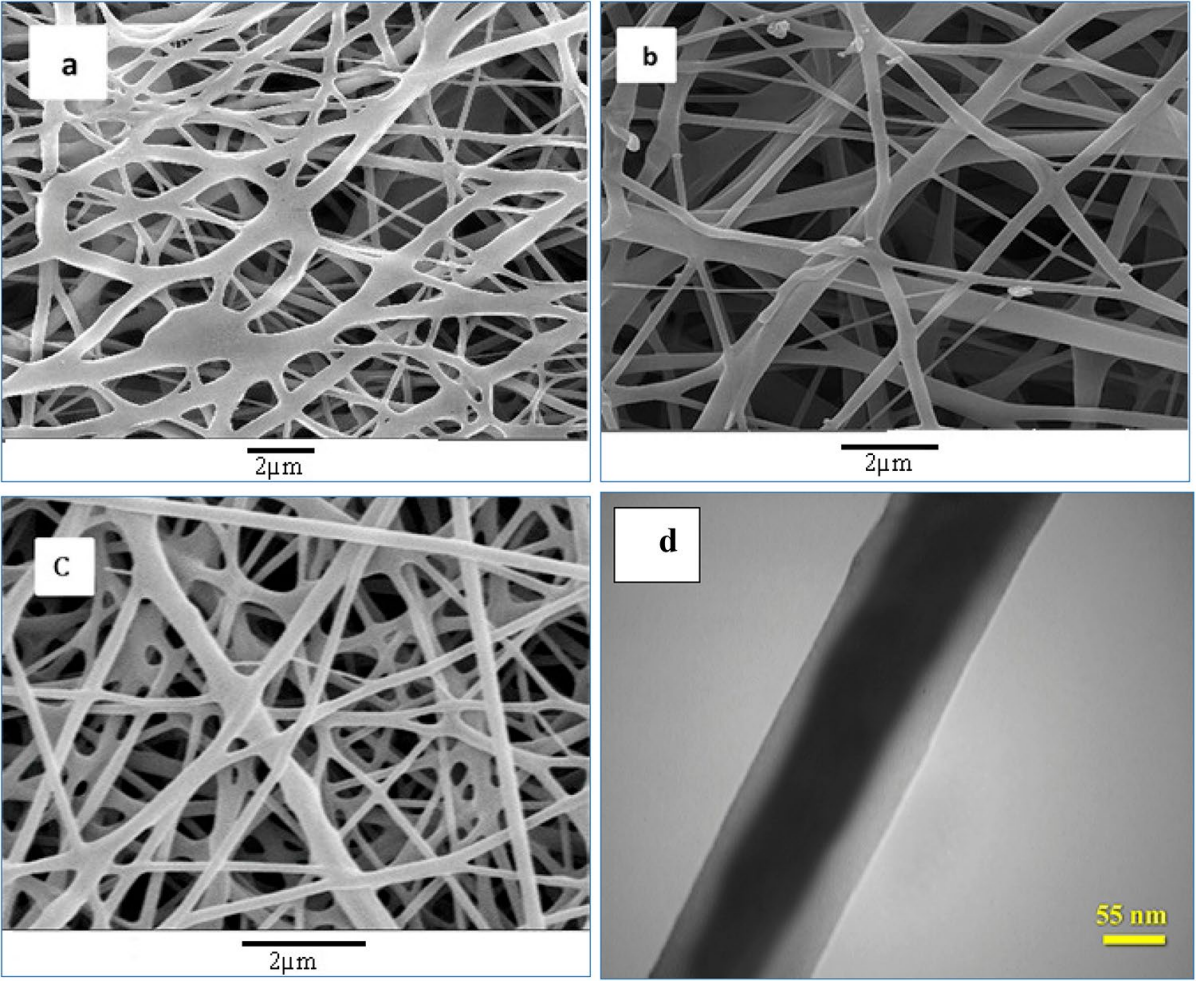
SEM image of (**a**) blend PEGDA/PU, (**b**) core/shell PEG/PU, (**c**) core/shell PU/PEGDA and (**d**) TEM image of core/shell PU/PEGDA.

In order to study the morphology of PEG/PU nanofibers, SEM images were utilized. As Fig. 5a–c indicates, all the fibers, including crosslinked blend PEGDA/PU, core/shell PEG/PU, and crosslinked core/shell PU/PEGDA nanofibers had a smooth surface. In order to confirm the core/shell structure of the fibers, TEM image was used. As shown in Fig. 5d, the structure of PU/PEGDA nanofiber is core/shell.

### Study of PEG/PU nanofibers and drug interaction

In order to investigate the interaction between meloxicam and PEG/PU nanofibers, FTIR analyses were utilized. Regarding the FTIR spectrum of meloxicam (Fig. S5a), peaks that appeared at 1153 cm^−1^, and 3290 cm^−1^ correspond to N–H and S=O stretching vibrations, respectively. Additionally, concerning the FTIR spectrum of pure core/shell PEG/PU (see Fig. S5b), peaks at 963, 1105, 1342, 2881, and 3357 cm^−1^ could be related to bending vibrations of CH_2_OH and stretching vibrations of C–O, C–H, and O–H, respectively. As the FTIR spectrum of core/shell PEG/PU with drug (Fig. S5c) reveals, there is an overlap between the peaks of drug and core/shell PEG/PU, which means there is no interaction between them and meloxicam was trapped in nanofiber’s structure. However, it should be mentioned due to low amount of the drug (0.1%) in fibers, it may not be detected using FT-IR analysis.

### Study of PEGDA synthesis and crosslinked structure of core/shell PU/PEGDA

C^13^NMR spectrum was applied to investigate the polyethylene glycol diacrylate synthesis. Looking at the C^13^NMR spectrum (see Fig. S6 in supplementary data) reveals that the peaks at 127.6 ppm, 132.6 ppm, and 169 ppm could be attributed to terminal acrylate groups, which confirms that the synthesis was carried out successfully.

Additionally, FTIR spectra of PEGDA and crosslinked PU/PEGDA were compared to confirm the crosslinked structure of core/shell PU/PEGDA. Regarding non-crosslinked PEGDA (see Fig. S6 in supplementary data), the peak at 1650 cm^−1^ is related to the double bond in the PEGDA structure, while this peak experienced a decrease in crosslinked PU/PEGDA spectrum, which proves crosslinking in the PU/PEGDA structure.

### In vitro release study of PEG/PU nanofibers

**Figure 6:**
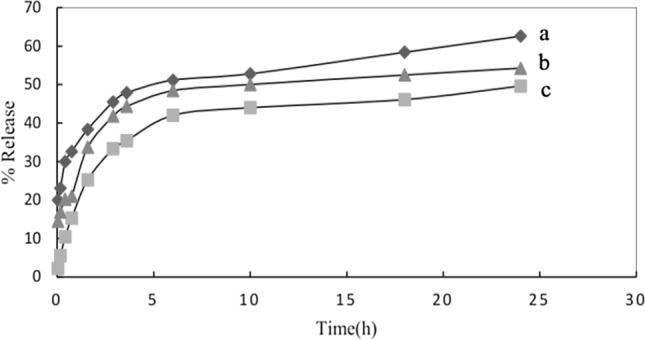
In vitro drug release profile of (a) blend PEGDA/PU (b) core/shell PEG/PU and (c) core/shell PU/PEGDA nanofibers.

The amount and rate of drug release from the nanofibers structure were investigated (see Fig. 6). Regarding core/shell PEG/PU (Fig. 6b), it showed a more controlled release in comparison with blend PEGDA/PU (Fig. 6a) and crosslinked core/shell PEG/PU-PGDA (Fig. 6c). To put it more tangibly, the core/shell structure moderates the buffer diffusion and drug conveyance, while for the blend PEGDA/PU, releasing happens faster. On the other hand, as regards crosslinked core/shell PEG/PU-PGDA, crosslinking with acrylate groups increased hydrophilicity which leads to a rise in the release rate.

### Study of release kinetics forPEG/PU nanofibers

Kinetics studies were carried out for different PEG/PU nanofibers, using the zero order, first order, Higuchi, and Korsmeyer-Peppas models. For this purpose, the obtained data were fitted and investigated using correlation coefficient R^2^. Studies were accomplished for blend PEGDA/PU, core/shell PEG/PU, and crosslinked core/shell PU/PGDA.

Figures S7, S8, and Table S2 show data for blend PEGDA/PU nanofibers and reveal that there is a fit to Higuchi with a correlation coefficient (R^2^) of 0.9312 for the first 45 min, while after 45 min to 6 h, it experienced a close fit to the Korsmeyer-Peppas model with a correlation coefficient (R2) of 0.9780. Furthermore, the controlling mechanisms, based on “n” values of 0.9084 and 0.234, a non-Fickian mechanism for the first 45 min and a Fickian mechanism from 45 min to 6 h were experienced, respectively. Moreover, concerning core/shell PEG/PU (see Figs. S9, S10, and Table S2), the Korsmeyer-Peppas model gave the closest fit with the R^2^ values of 0.9983 and 0.9898 for both the first 45 min and after 45 min, respectively. Besides, as regards the mechanism controlling drug release, meloxicam experienced a non-Fickian mechanism for the first 45 min and a Fickian mechanism after 45 min to 6 h with the “n” value of 0.6157 and 0.3801, respectively.

**Figure 7:**
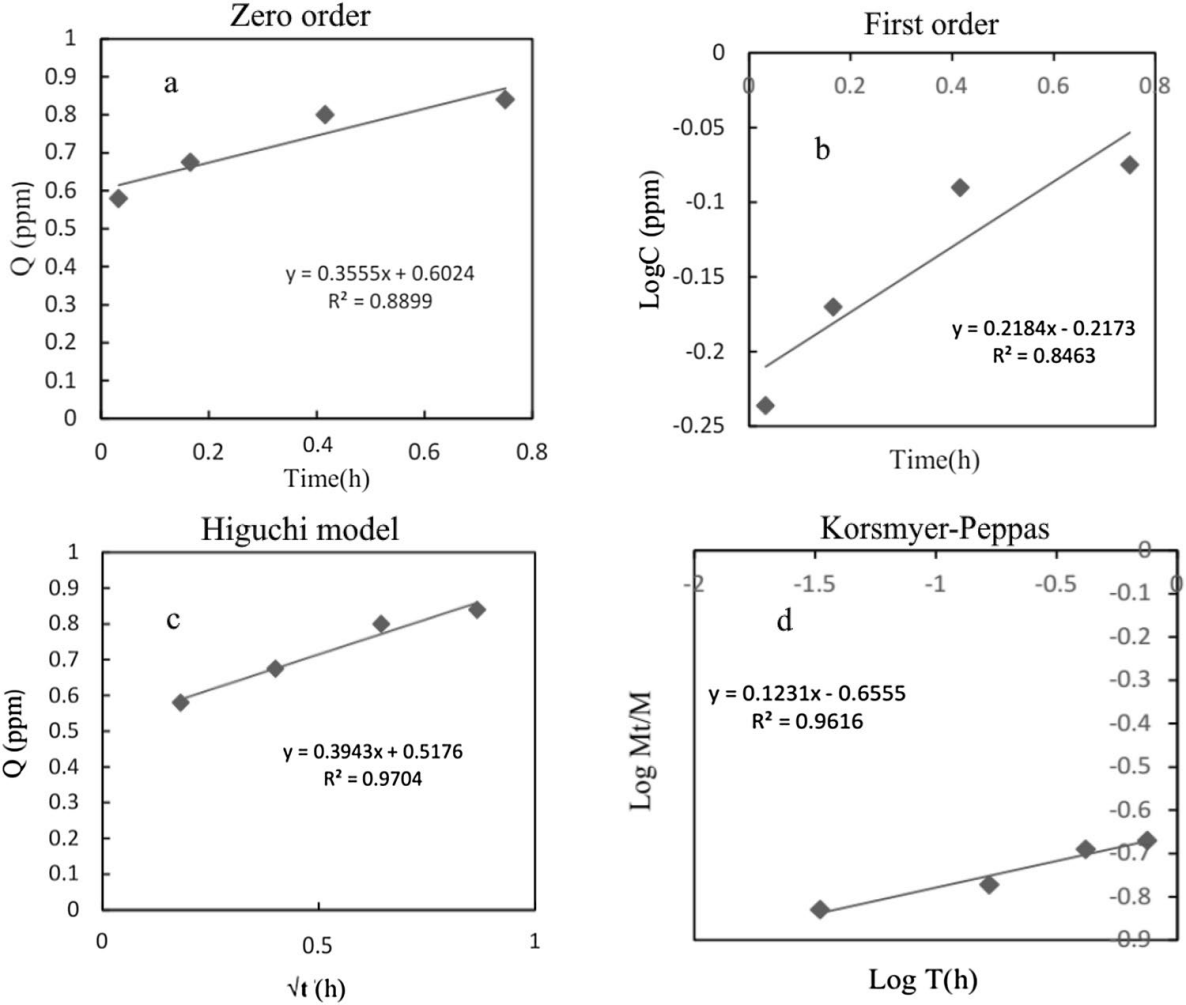
Evaluated kinetic models for meloxicam release over first 45 min from core/shell PU/PEGDA (**a**) Zero order, (**b**) First order, (**c**) Higuchi and (**d**) Korsmeyer-Peppas.

On the other hand, crosslinked core/shell PEG/PU-PGDA has the closest fits to the Higuchi model for the first 45 min with the R^2^ value of 0.9704 and Korsmeyer-Peppas after 45 min to 6 h with the R^2^ value of 0.9630. Additionally, the controlling mechanism according to the value of “n,” with the amounts of 0.1231 and 0.2733 for the first 45 min and after 45 min to 6 h, respectively, was proved to be a Fickian mechanism (see Figs. 7, S11, and Table S3).

### XRD analyses of core/shell PEG/PU and meloxicam

XRD patterns for core/shell PEG/PU and meloxicam are shown in Fig. S12. As it could conspicuously be observed, the diffraction peaks at 2θ of 26.0552°, 22.1595°, 19.43°, 18.78°, 15.12°, 13.24° and 6.689° show that meloxicam has a crystal structure. Besides, as concerns core/shell PEG/PU, peaks appeared at 19°, 21°, and 23 could be attributed to PEG and reveals that PU has an amorphous structure in the nanofiber.

### DSC analyses of core/shell PEG/PU and meloxicam

DSC analyses were used in order to study the morphology of Meloxicam in the nanofibers’ structure. For this purpose, DSC analyses were accomplished for meloxicam, pure core/shell PEG/PU, and core/shell PEG/PU with the drug. As results show (see Fig. S13 in Supplementary data), meloxicam has a crystal structure and starts melting at the temperature of 267 °C, and a sharp peak is observed at this point. In addition, the peak that appeared at 64 °C corresponds to the PEG melting point. Comparing the graphs of core/shell PEG/PU with drug, meloxicam, and pure core/shell PEG/PU reveals that there is a shift in the peaks’ positions, which means there is an interaction between the drug and nanofiber. In fact, the peaks appeared at 285 °C, and 66 °C are attributed to the meloxicam melting point and show its crystal structure in nanofiber.

### Swelling study of the nanofibers

**Figure 8:**
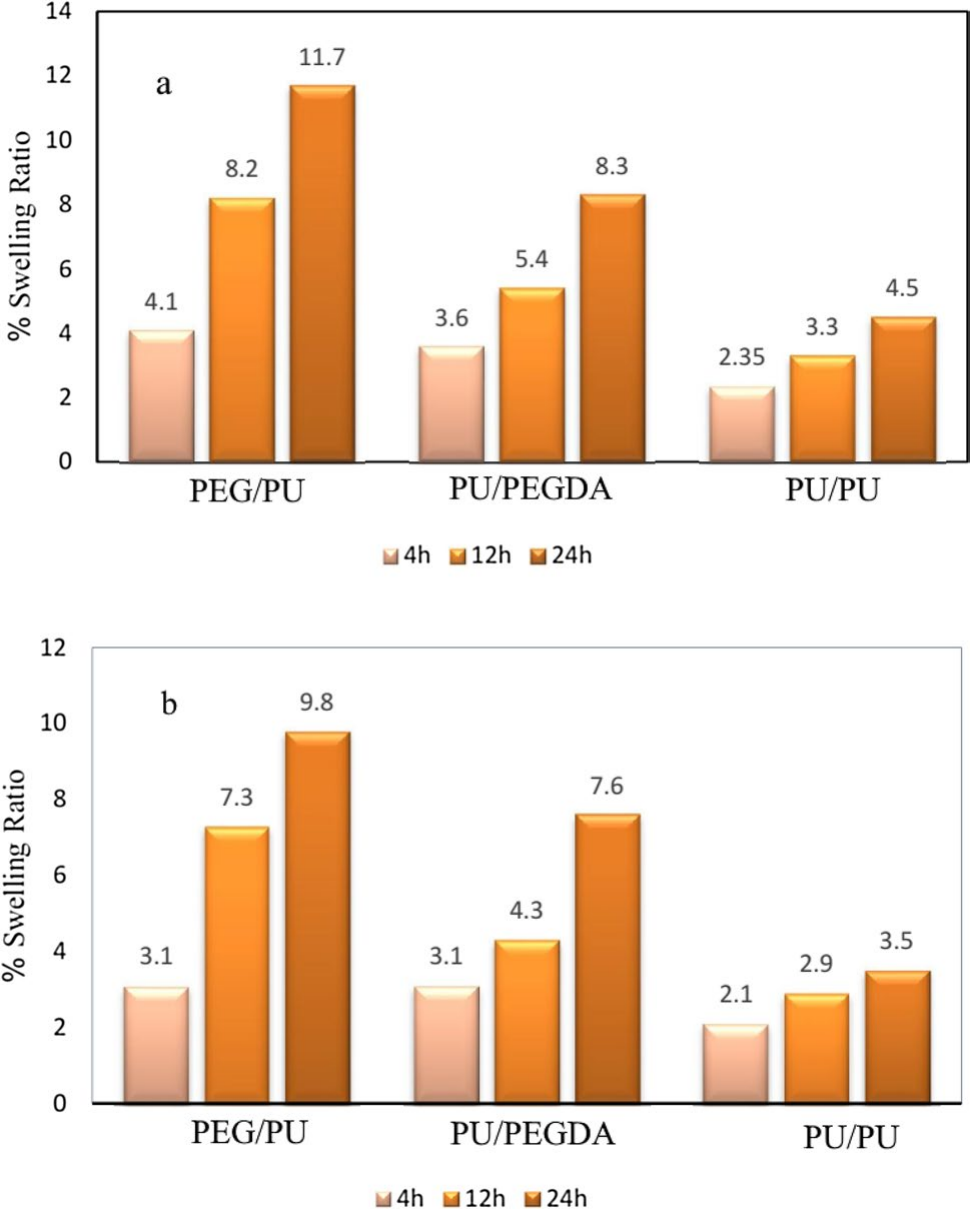
Swelling measurement of (**a**) pure nanofibers and (**b**) nanofibers with drug.

Studies have shown that swelling is one of the most critical factors in drug delivery. Accordingly, swelling measurement was carried out for nanofibers. As results show, core/shell PEG/PU had the highest amount of swelling degree due to hydrophilic PEG groups in its structure, while crosslinked PU/PEGDA had lower swelling, and this can be concluded that crosslinking decreased the swelling degree for this nanofiber. On the other hand, PU/PU nanofiber showed the least amount of swelling (see Fig. 8). On the other hand, as shown in Fig. 8, the rate and amount of water absorption has increased in samples containing ethylene glycol groups. The presence of ethylene glycol groups in the structure of the PU/PEGDA and PEG/PU fibers makes them more hydrophilic than the PU/PU fiber. Also, the presence of the hydroxyl functional group in the PEG/PU fiber makes it more hydrophilic than the PU/PEGDA fiber. In addition, the presence of meloxicam drug molecule between the polymer chains of all three carriers has reduced their water absorption and hydrophilicity, which can be related to (1) the hydrophobic structure of meloxicam and (2) the interaction between the hydroxyl groups of meloxicam and the hydrophilic structures of the carriers. Establishing a hydrogen bond between the meloxicam and the carriers will reduce the tendency of the carriers to interact with water, which results in a decrease in the rate and amount of water absorption in carriers containing drugs (Fig. 8b) compared to carriers without drugs (Fig. 8a).

## Conclusion

Applying an in situ photopolymerization reaction the PEG was crosslinked during electrospinning. The Light curable PEGDA was synthesized and utilized as a modifier. The results obtained from 1H NMR, 13C NMR and FT-IR analysis showed that PEGDA has successfully been synthesized. Different meloxicam drug carrier nanofibers such as monolithic, blend and core/shell nanofibers were synthesized through electrospinning technique by using PU and PEG polymers. According to the results obtained from the FTIR analysis of the double bonds (the band intensity has declined sharply at 1637 cm^−1^), it has been found that the electrospun fibers have successfully been crosslinked with visible light radiation during electrospinning. The obtained results showed that the presence of ethylene functional groups, especially core/shell PU/PEGDA nanofibers, in the structure of fibers reduces and controls the drug release. So this new one-step method uses an in situ photopolymerization reaction can be used to design the nanofibers drug delivery system without dissolution and destroying the nanofibers during the crosslinking.

## Supplementary Information

Supplementary Information.

## Data Availability

All data associated with this study are present in the paper.
