# Supplementary material for: Applying in-situ visible photopolymerization for fabrication of electrospun nanofibrous carrier for meloxicam delivery
Source: Sci Rep. 2023 Jun 16;13:9741. doi: 10.1038/s41598-023-36893-9 (PMC10275867; doi:10.1038/s41598-023-36893-9)
Supplement: Supplementary file 1 — Supplementary Information. [file 41598_2023_36893_MOESM1_ESM.docx]

**Supplementary material**

**Applying in-situ visible photopolymerization for fabrication of electrospun nanofibrous carrier for meloxicam delivery**

Z. Ahmadipour, M. S. Seyed Dorraji^*^, H. R. Ashjari, F. Dodangeh, M. H. Rasoulifard

Applied Chemistry Research laboratory, Department of Chemistry, Faculty of Science, University of Zanjan, Zanjan, Iran.

*: corresponding author

E-mail: dorraji@znu.ac.ir

Tel & Fax: +98 24 3305 2477

Table S1. Mathematical modeling of meloxicam release from monolithic PU nanofibers and core/shell PU/PU nanofibers

| **Monolithic PU Nanofibers** | | | | | |
| --- | --- | --- | --- | --- | --- |
| Time  45 min | Model | K | n | R^2^ | Mechanism |
| <45 min | zero Order | 3.3427 | - | 0.9821 | - |
|  | First order | -2.4480 | - | 0.8550 | - |
|  | Higuchi | 3.5777 | - | 0.9968 | - |
|  | Korsmeyer-Peppas | 0.63576 | 0.5904 | 0.9976 | Non-Fickian |
| 45min-6h | zero Order | 0.1415 | - | 0.9512 | - |
|  | First order  45 min- 6 h | -0.0449 | - | 0.9365 | - |
|  | Higuchi | 0.5402 | - | 0.9857 | - |
|  | Korsmeyer-Peppas | 0.5356 | 0.1522 | 0.9986 | Fickian Mechanism |
| **Core/shell PU/PU Nanofibers** | | | | | |
| Time  45 min | Model | K | n | R^2^ | Mechanism |
| <45 min | zero Order | 1.6979 | - | 0.9393 | - |
|  | First order | -4.3102 | - | 0.9956 | - |
|  | Higuchi | 0.9661 | - | 0.7974 | - |
|  | Korsmeyer-Peppas | 0.3790 | 0.5772 | 0.9947 | Non-Fickian |
| 45min-6h | zero Order | 0.2603 | - | 0.8991 | - |
|  | First order  45 min- 6 h | -0.0987 | - | 0.8511 | - |
|  | Higuchi | 6.6972 | - | 0.9627 | - |
|  | Korsmeyer-Peppas | 0.3480 | 0.3820 | 0.9823 | Fickian Mechanism |

| **Blend PEG/PU Nanofibers** | | | | | |
| --- | --- | --- | --- | --- | --- |
| Time  45 min | Model | k | n | R^2^ | Mechanism |
| <45 min | zero Order | 1.0246 | - | 0.9119 | - |
|  | First order | -0.6577 | - | 0.8908 | - |
|  | Higuchi | 0.5524 | - | 0.9312 | - |
|  | Korsmeyer-Peppas | 0.6223 | 0.9084 | 0.8739 | Non-Fickian |
| 45min-6h | zero Order | 0.1701 | - | 0.8831 | - |
|  | First order | -0.0628 | - | 0.8572 | - |
|  | Higuchi | 0.6615 | - | 0.9442 | - |
|  | Korsmeyer-Peppas | 0.3496 | 0.2340 | 0.9780 | Fickian Mechanism |
| **Core/shell**  **PEG/PU Nanofibers** | | | | | |
| Time  45 min | Model | k | n | R^2^ | Mechanism |
| <45 min | zero Order | 0.4369 |  | 0.9891 | - |
|  | First order | -2.279 |  | 0.8959 | - |
|  | Higuchi | 0.4646 |  | 0.9911 | - |
|  | Korsmeyer-Peppas | 0.1783 | 0.6157 | 0.9983 | Non-Fickian |
| 45min-6h | zero Order | 0.0811 |  | 0.903 | - |
|  | First order  45 min- 6 h | -0.0976 |  | 0.8266 | - |
|  | Higuchi | 0.3141 |  | 0.9578 | - |
|  | Korsmeyer-Peppas | 0.2164 | 0.3801 | 0.9898 | Fickian Mechanism |

Table S2. Mathematical modeling of meloxicam release from blend PEG/PU nanofibers and core/shell PEG/PU nanofibers

45 min- 6 h

Table S3. Mathematical modeling of meloxicam release from core/shell PU/PEGDA nanofibers

45 min

| **Time** | **Model** | **k** | **n** | **R^2^** | **Mechanism** |
| --- | --- | --- | --- | --- | --- |
| <45 min | zero Order | 0.3555 |  | 0.8899 | - |
|  | First order | -0.5029 |  | 0.8463 | - |
|  | Higuchi | 0.3943 |  | 0.9704 | - |
|  | Korsmeyer-Peppas | 0.2210 | 0.1231 | 0.9616 | Fickian Mechanism |
| 45min-6h | zero Order | 0.1258 |  | 0.8914 | - |
|  | First order  45 min- 6 h | -0.0762 |  | 0.8533 | - |
|  | Higuchi | 0.4884 |  | 0.9500 | - |
|  | Korsmeyer-Peppas | 0.3050 | 0.0.2733 | 0.9630 | Fickian Mechanism |


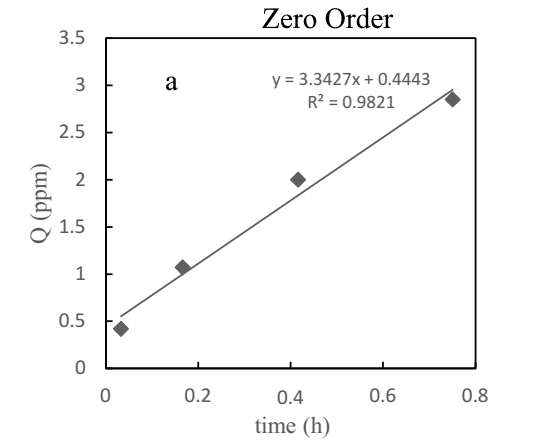

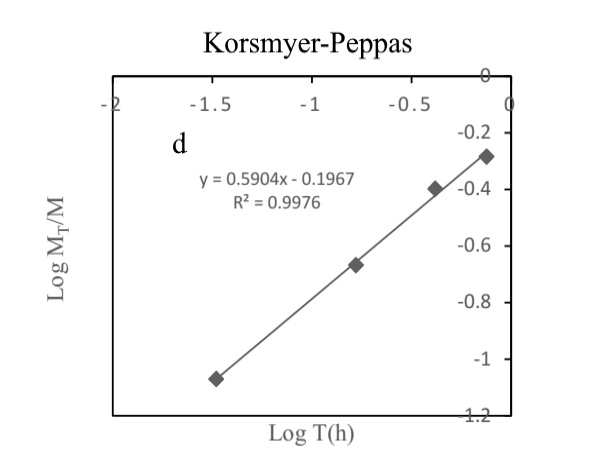

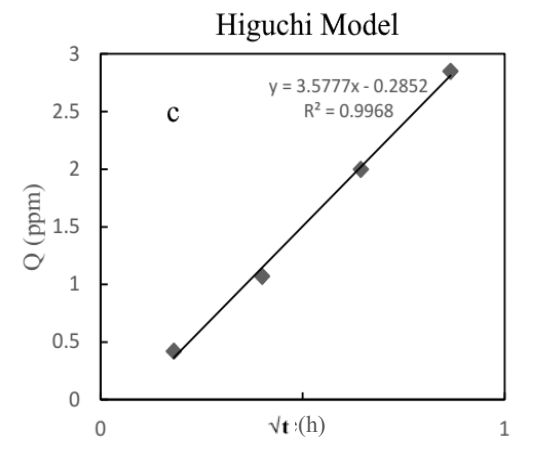

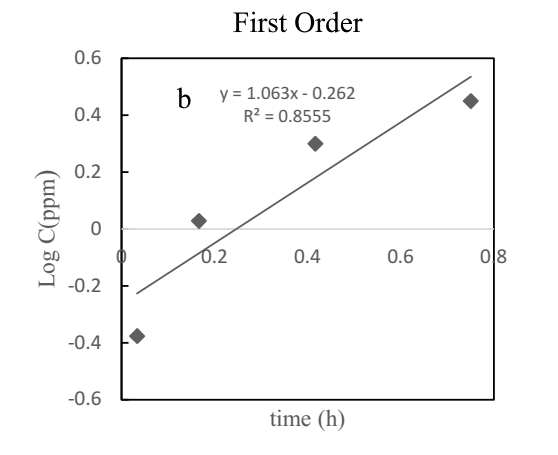


Fig. S1. Evaluated kinetic models for meloxicam release over the first 45 min from monolithic PU (a) Zero order, (b) First order, (c) Higuchi and (d) Korsmeyer-Peppas


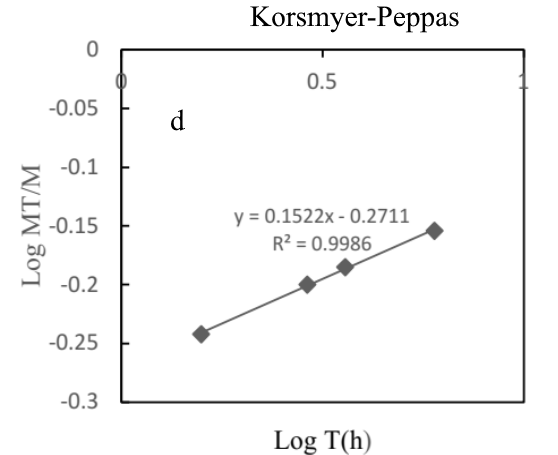

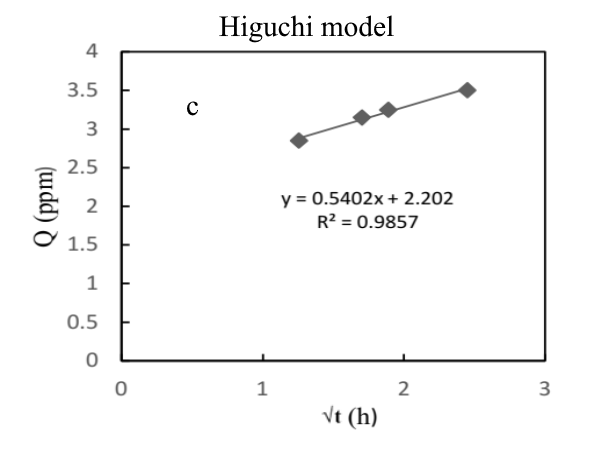

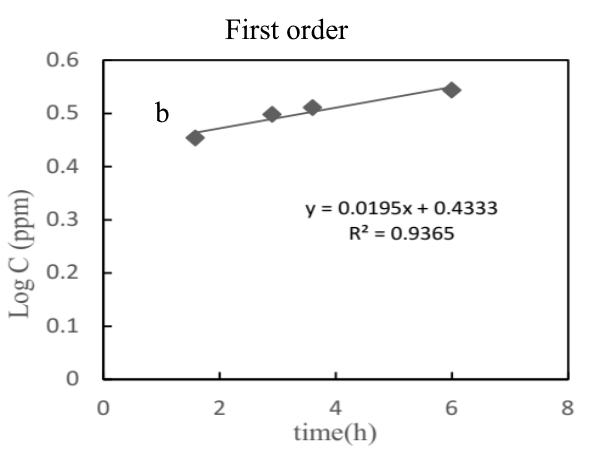

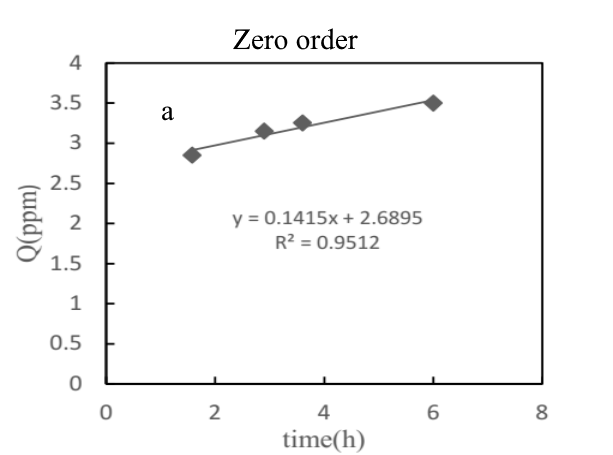


Fig. S2. Evaluated kinetic models for meloxicam release after 45 min from monolithic PU (a) Zero order, (b) First order, (c) Higuchi and (d) Korsmeyer-Peppas


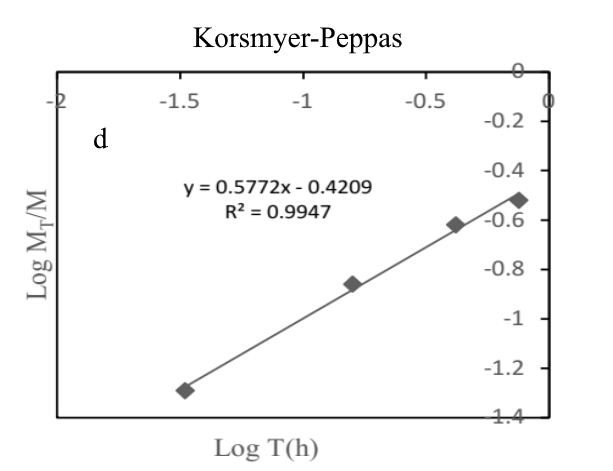

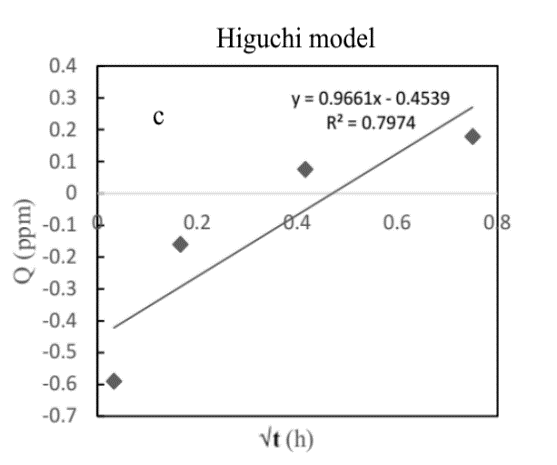

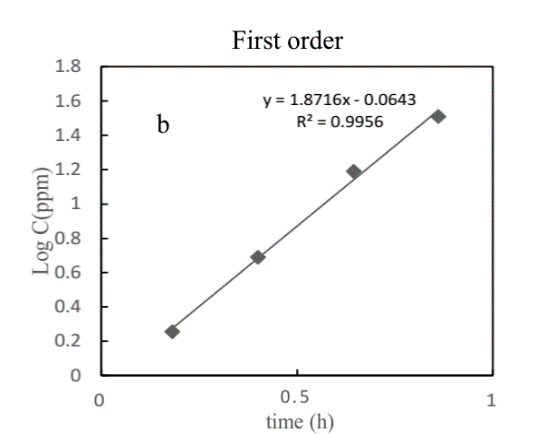

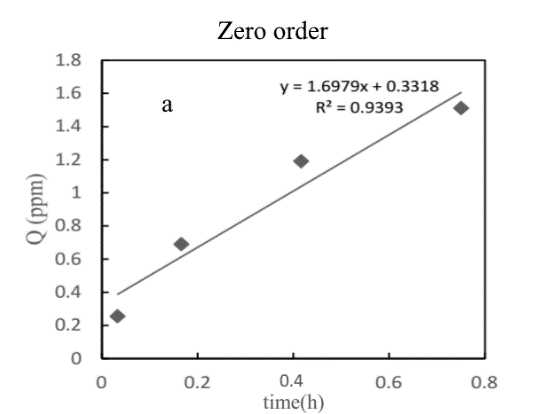


Fig. S3. Evaluated kinetic models for meloxicam release over the first 45 min from core/shell PU/PU (a) Zero order, (b) First order, (c) Higuchi and (d) Korsmeyer-Peppas


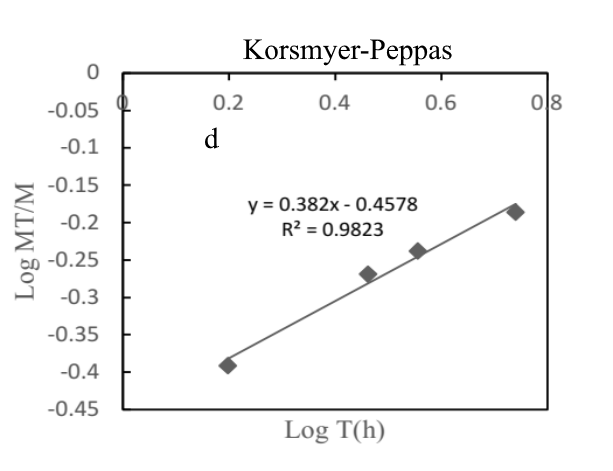

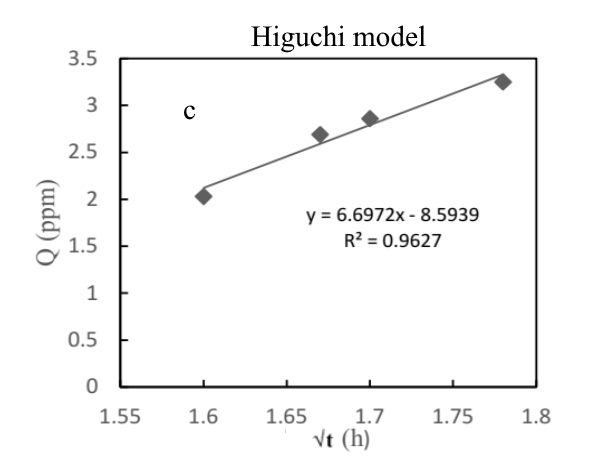

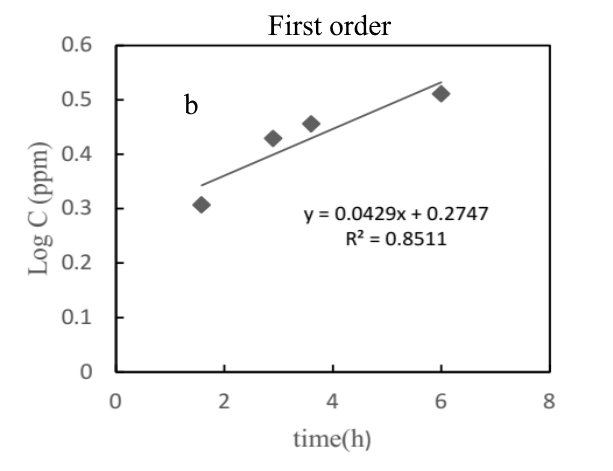

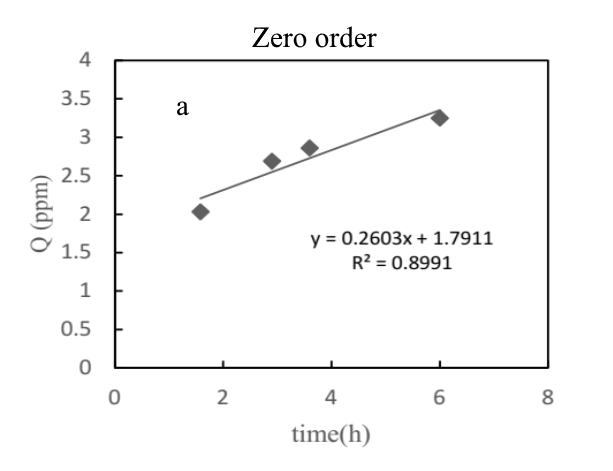


Fig. S4. Evaluated kinetic models for meloxicam release after 45 min from core/shell PU/PU (a) Zero order, (b) First order, (c) Higuchi and (d) Korsmeyer-Peppas


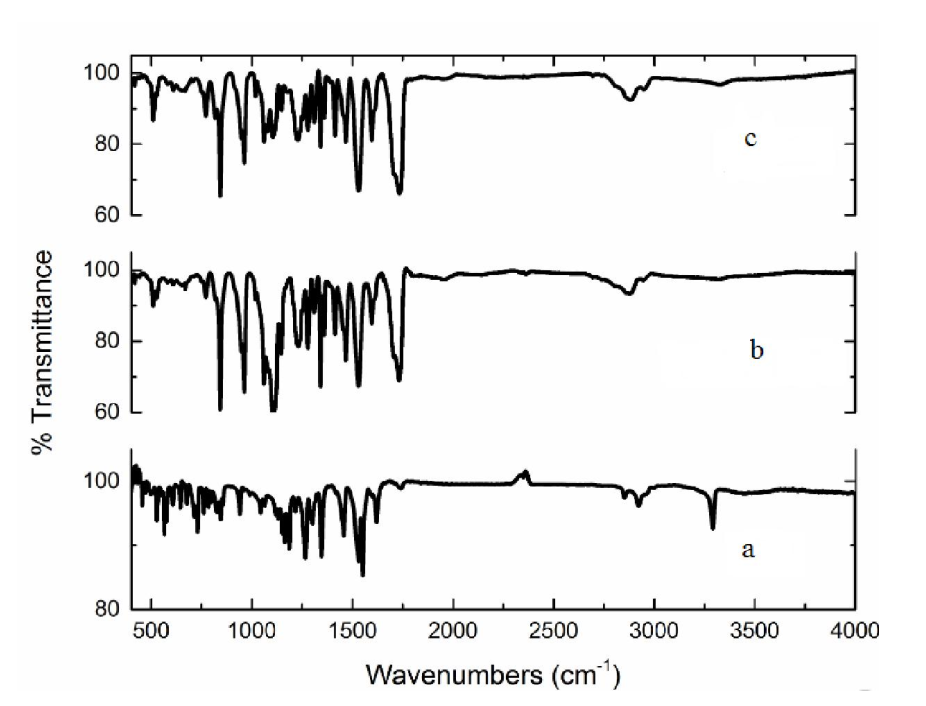
Fig. S5. Analysis of chemical structure of (a) meloxicam (b) pure core/shell PEG/PU (c) core/shell PEG/PU with drug


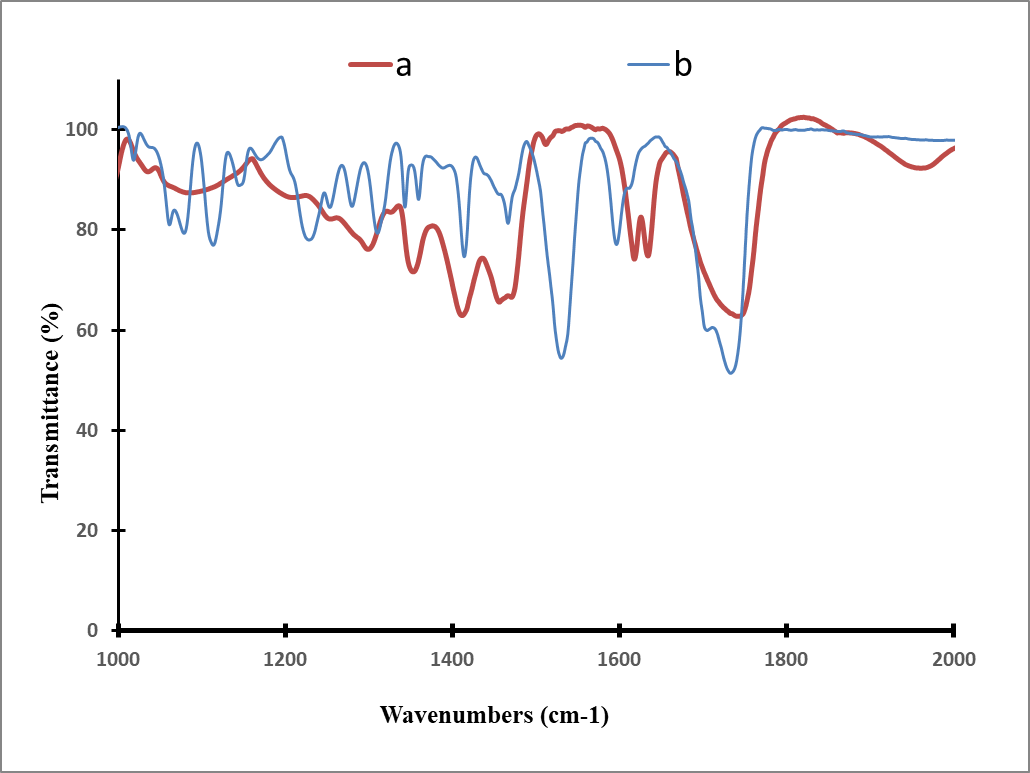


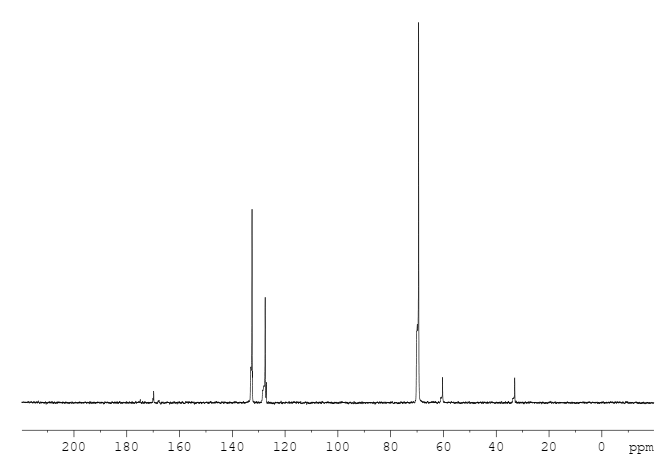


c

Fig. S6. Analysis of chemical structure of (a) noncross-linked PEGDA (b) crosslinked core/shell PU/PEGDA and (c) PEGDA


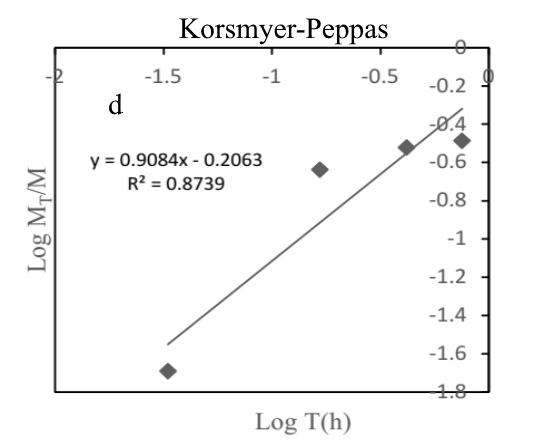

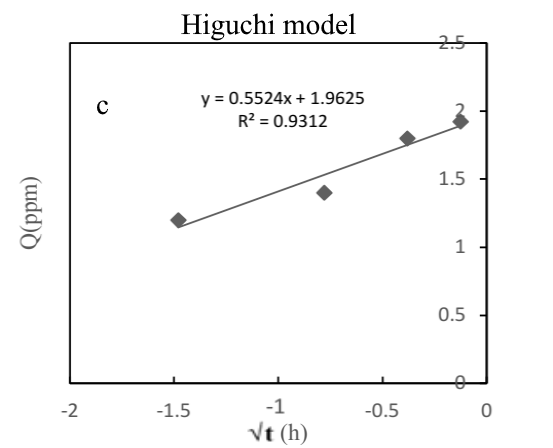

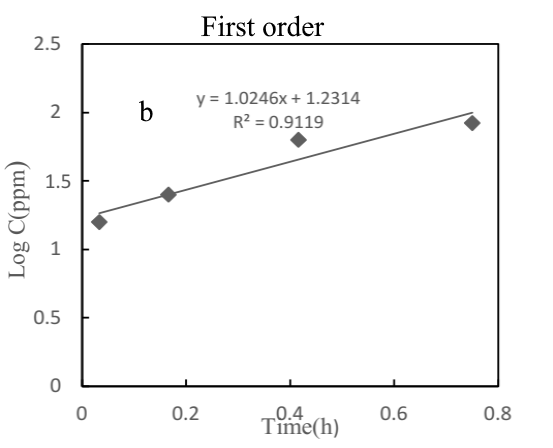

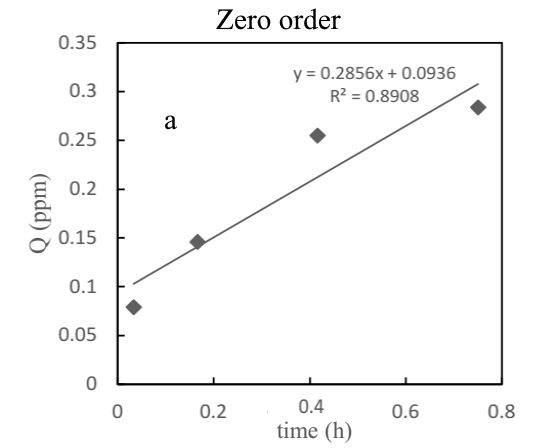


Fig. S7. Evaluated kinetic models for meloxicam release over first 45 min from blend PEG/PU (a) Zero order, (b) First order, (c) Higuchi and (d) Korsmeyer-Peppas


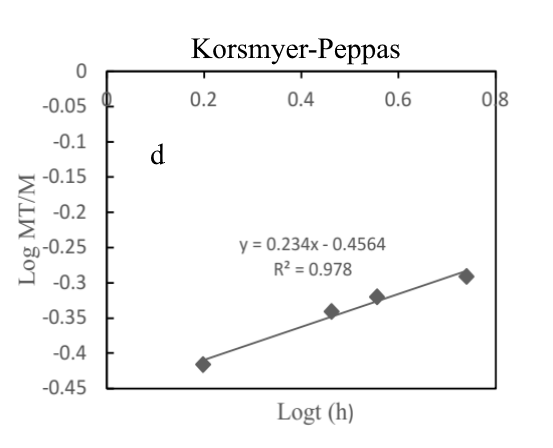

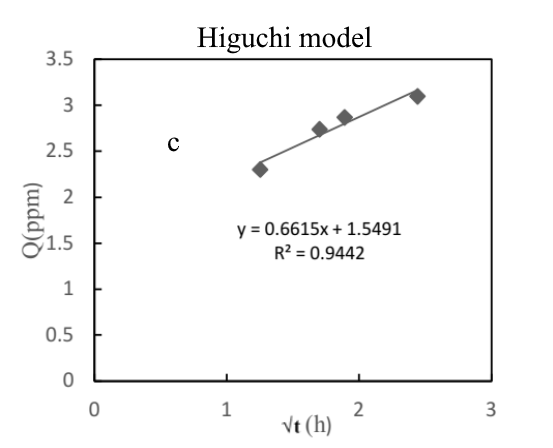

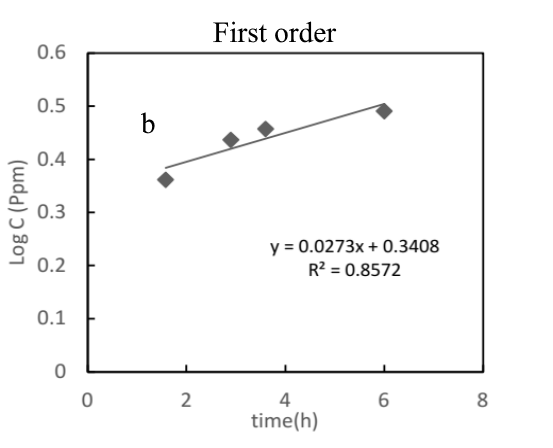

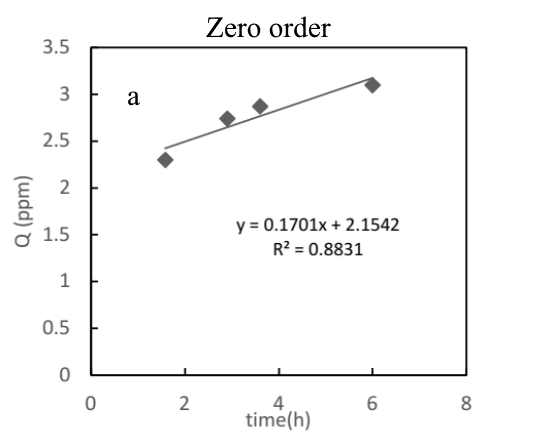


Fig. S8. Evaluated kinetic models for meloxicam release after 45 min from blend PEG/PU (a) Zero order, (b) First order, (c) Higuchi and (d) Korsmeyer-Peppas


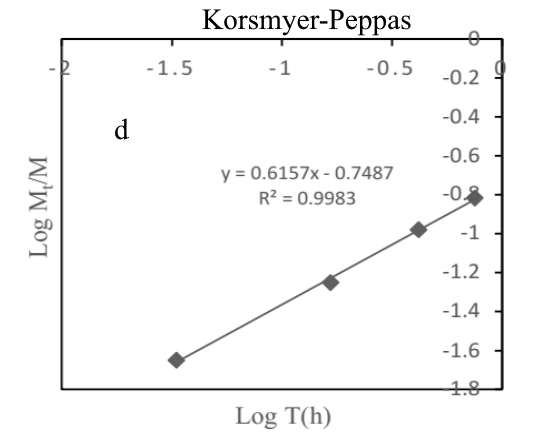

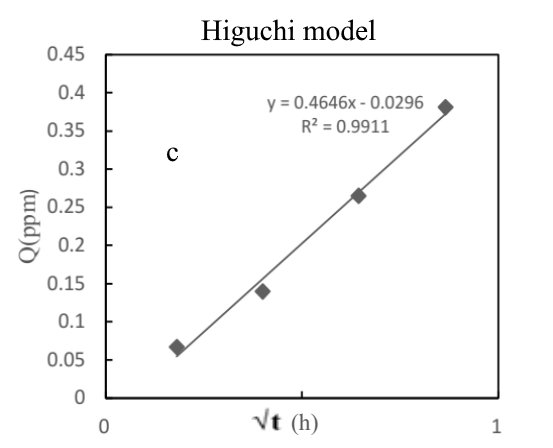

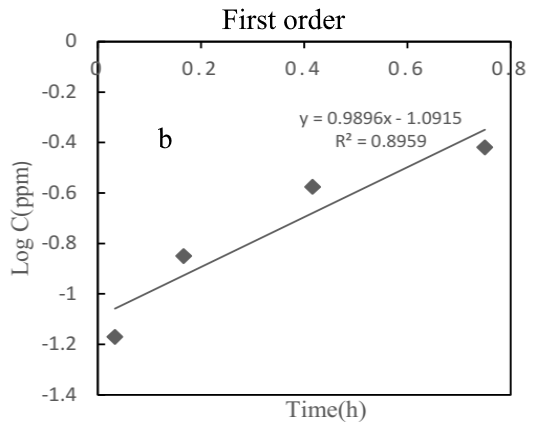

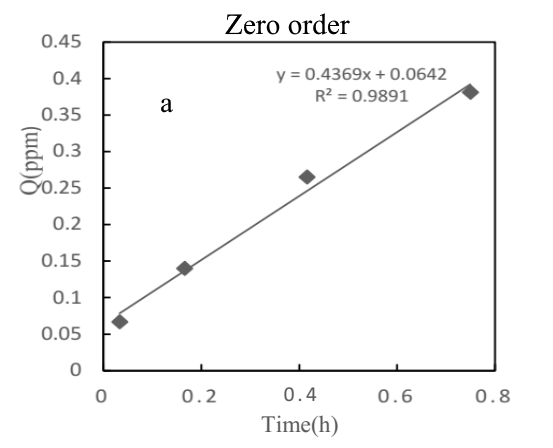


Fig. S9. Evaluated kinetic models for meloxicam release over first 45 min from core/shell PEG/PU (a) Zero order, (b) First order, (c) Higuchi and (d) Korsmeyer-Peppas


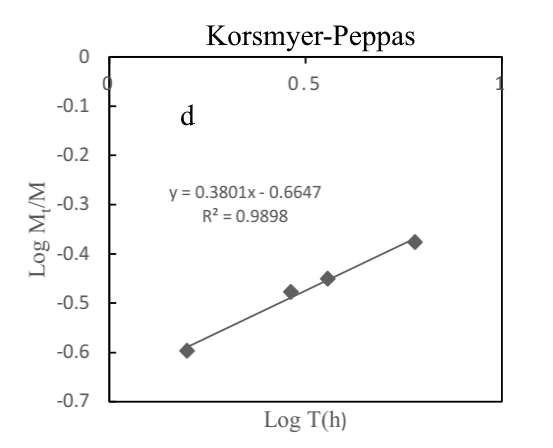

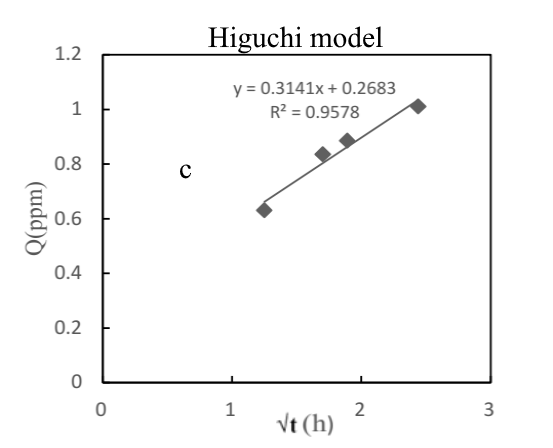

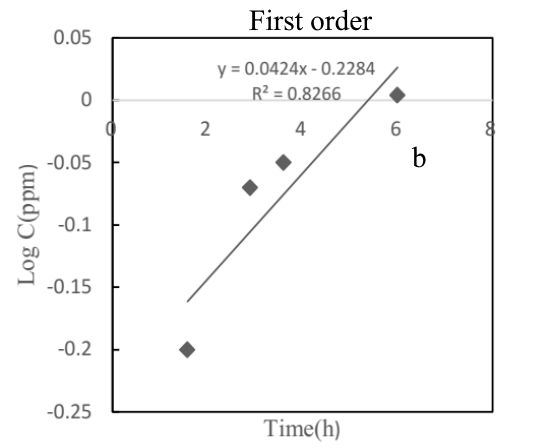

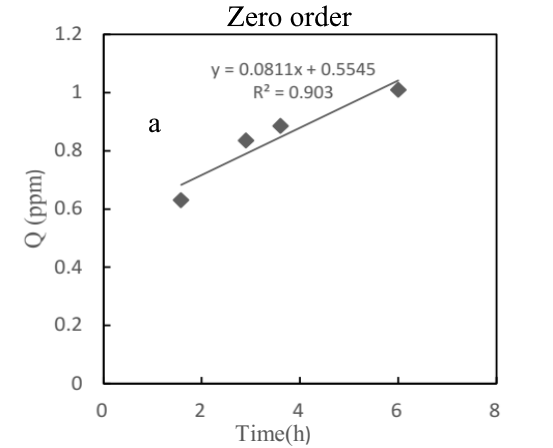
 Fig. S10. Evaluated kinetic models for meloxicam release after 45 min from core/shell PEG/PU (a) Zero order, (b) First order, (c) Higuchi and (d) Korsmeyer-Peppas


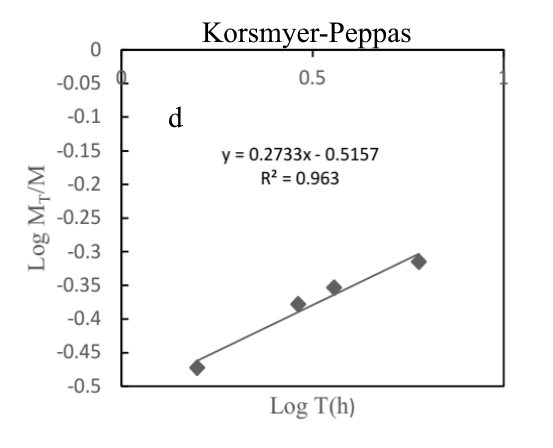

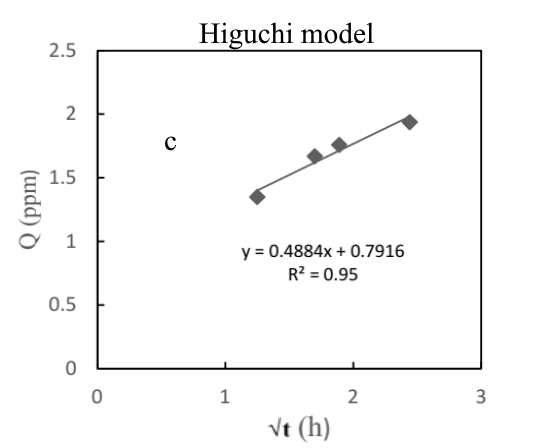

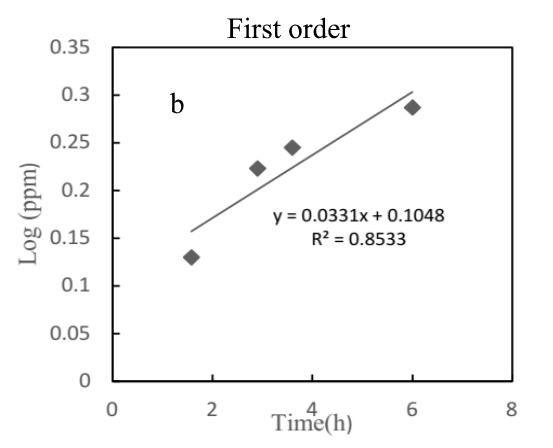

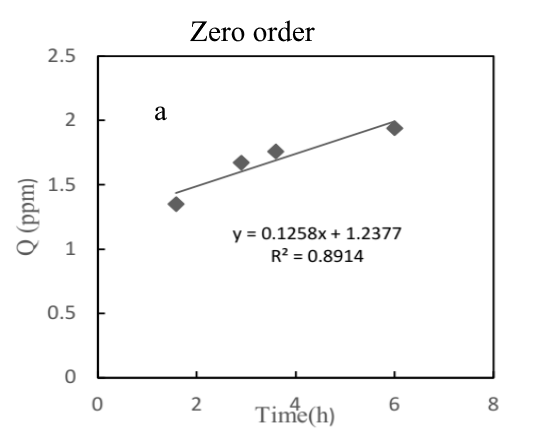


Fig. S11. Evaluated kinetic models for meloxicam release after 45 min from core/shell PU/PEGDA (a) Zero order, (b) First order, (c) Higuchi and (d) Korsmeyer-Peppas


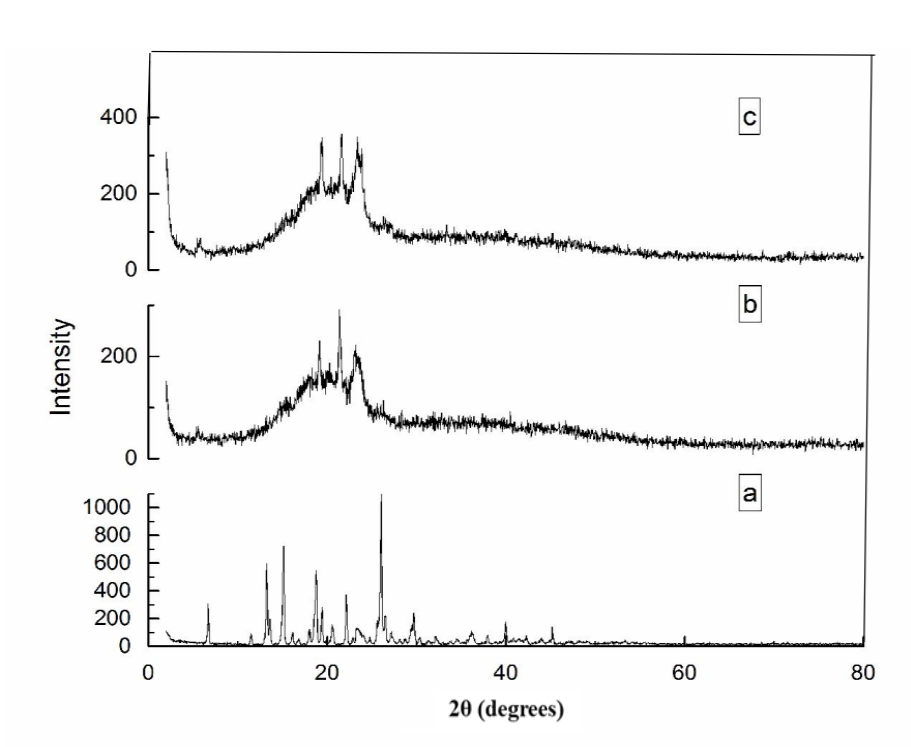


Fig. S12.XRD pattern analysis of, (a) meloxicam (b) pure core/shell PEG/PU and (c) core/shell PEG/PU with drug


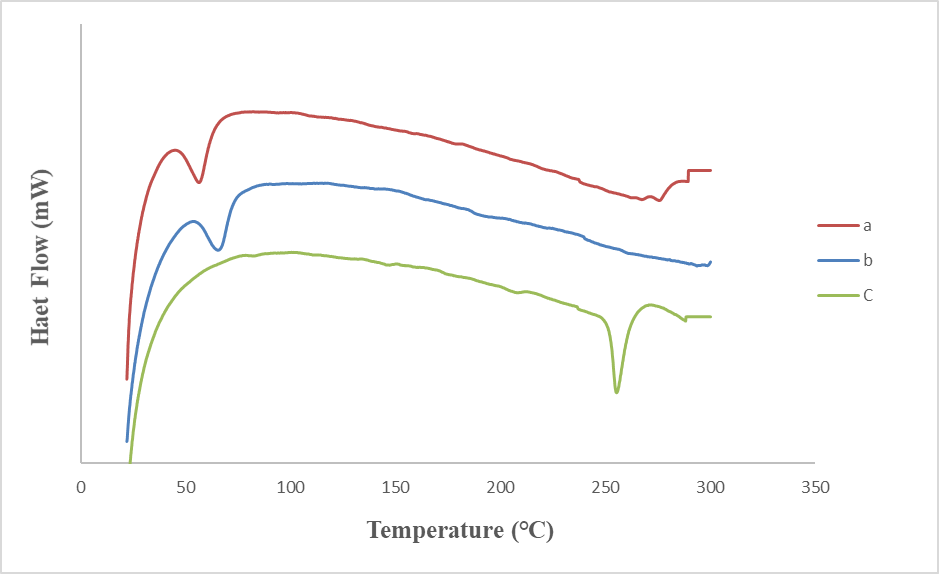


Fig.13. DSC analyses of (a) pure core/shell PEG/PU, (b) core/shell PEG/PU with drug and (c) meloxicam
